# Both Acute and Consecutive Days of Formoterol Stimulation Influence Myogenic, Mitochondrial, and myomiR Gene Expression in Human Skeletal Muscle Cells

**DOI:** 10.3390/muscles2010008

**Published:** 2023-02-22

**Authors:** Ryan A. Gordon, Emily L. Zumbro, Gena D. Guerin, Matthew L. Sokoloski, Vic Ben-Ezra, Christopher S. Brower, Rhett B. Rigby, Anthony A. Duplanty

**Affiliations:** 1 Department of Biology, Drury University, Springfield, MO 65802, USA; 2 Department of Medicine, University of Alabama at Birmingham, Birmingham, AL 35294, USA; 3 Department of Kinesiology, Saginaw Valley State University, Saginaw, MI 48710, USA; 4 School of Health Promotion & Kinesiology, Texas Woman’s University, Denton, TX 76204, USA; 5 Department of Biology, Texas Woman’s University, Denton, TX 76204, USA

**Keywords:** skeletal muscle, myogenesis, exercise mimetic, miRNA, growth

## Abstract

Skeletal muscle physiology is regulated by microRNA that are localized within skeletal muscle (myomiRs). This study investigated how the expression of myomiRs and genes regulating skeletal muscle mass and myogenesis are influenced in response to acute and consecutive days of exercise-related signaling using the exercise mimetic, formoterol, in vitro. Human skeletal muscle cells were proliferated and differentiated for 6 days. Experimental conditions included: (a) control, (b) acute formoterol stimulation (AFS), and (c) consecutive days of formoterol stimulation (CFS). For AFS, myotubes were treated with 30 nM of formoterol for three hours on day 6 of differentiation, and this was immediately followed by RNA extraction. For CFS, myotubes were treated with 30 nM of formoterol for three hours on two or three consecutive days, with RNA extracted immediately following the final three-hour formoterol treatment. We observed increased myomiR expression for both AFS and CFS. AFS appeared to promote myogenesis, but this effect was lost with CFS. Additionally, we observed increased expression of genes involved in metabolism, mitochondrial biogenesis, and muscle protein degradation in response to AFS. myomiR and gene expression appear to be sensitive to acute and long-term exercise-related stimuli, and this likely contributes to the regulation of skeletal muscle mass.

## 1. Introduction

Skeletal muscle is a dynamic tissue that exhibits remarkable plasticity in response to molecular and metabolic stimuli [1]. Fundamental to this plasticity is skeletal muscle’s ability to regenerate, which is mediated by myogenic satellite cells [2]. In addition to their role in regeneration, satellite cells contribute to skeletal muscle growth by increasing the myonuclear content of skeletal muscle in response to anabolic stimuli, thereby increasing the transcriptional and translational capacity of a skeletal muscle fiber [3,4]. Molecular regulation of satellite cells is multifaceted but is largely governed by a series of transcription factors, referred to as myogenic regulatory factors (MRFs). Initially, MRFs promote the activation and proliferation of these satellite cells into precursor cells (i.e., myoblasts), while later regulating the commitment of these myoblasts, through differentiation and maturation, into mature myotubes. This process, referred to as myogenesis, encompasses the role satellite cells have in the regeneration and growth of skeletal muscle [5,6].

In addition to MRFs, myogenesis is regulated, in part, by microRNA (miRNA or miR) that are predominantly localized within skeletal muscle (myomiR) [7,8]. The myomiR family, which includes miR-1, miR-133a, miR-133b, miR-486, miR-499, miR-206, miR-208a, and miR-208b, influences myogenesis through interaction with MRFs, including myogenic differentiation 1 (MyoD) and myogenin (MyoG) [9,10]. During mid-stage myogenesis, miR-206 and miR-486 have important roles in promoting myoblast differentiation, both appearing to target and inhibit Pax7, a primary regulator of myoblast development during early-stage myogenesis [7,10]. Beyond myogenesis, the myomiR family also influences cell signaling pathways involved in the regulation of skeletal muscle growth. Again, miR-206 and miR-486 both contribute to this process by converging on Akt, with miR-206 directly inhibiting Akt. miR-486 indirectly promotes Akt expression [11], while also inhibiting Foxo1 expression [7]. Thus, both miRs have important roles in the regeneration and growth of skeletal muscle.

Exercise, particularly resistance exercise, is a potent stimulator of both myogenesis and skeletal muscle growth [7,12,13]. Importantly, myomiRs, mediating these skeletal muscle processes, are responsive to both acute and chronic exercise [14,15]. Despite this, our understanding of how the myomiRs influence skeletal muscle function and physiology at the molecular level, particularly in response to exercise, is still being determined.

To determine molecular responses to exercise stimuli, an exercise mimetic may be used in rodent and/or cell culture models. A variety of exercise mimetics have been proposed, with each commonly converging on one or more molecular signaling pathways [16]. Stimulating skeletal muscle through an exercise mimetic, specifically through the β2-adrenergic receptor (β2AR) pathway, can influence myoblast differentiation and myogenic activity [17]. Our lab has previously observed that formoterol, a β2AR-agonist, influences gene expression related to mitochondrial biogenesis, oxidative metabolism, and myogenesis in human skeletal muscle cells (unpublished findings). Considering this, we sought to gain a greater understanding of formoterol’s effects on skeletal muscle by determining how miR-206 and miR-486 gene expression, along with MRFs and regulators of skeletal muscle homeostasis, is affected in response to an exercise mimetic (i.e., formoterol) in human skeletal muscle cells.

## 2. Results

This study utilized an in vitro model of human skeletal muscle myogenesis to determine how the exercise mimetic, formoterol, may affect the gene expression of miRNA, MRFs, and proteins involved in the regulation of skeletal muscle growth and function. For simplicity, the results from this study are organized into the following categories of genes related to: (a) myogenesis, (b) skeletal muscle growth, (c) cell and mitochondrial biogenesis, (d) miRNA.

### 2.1. Formoterol Stimulates Gene Expression of Myogenic Regulatory Factors

**Figure 1:**
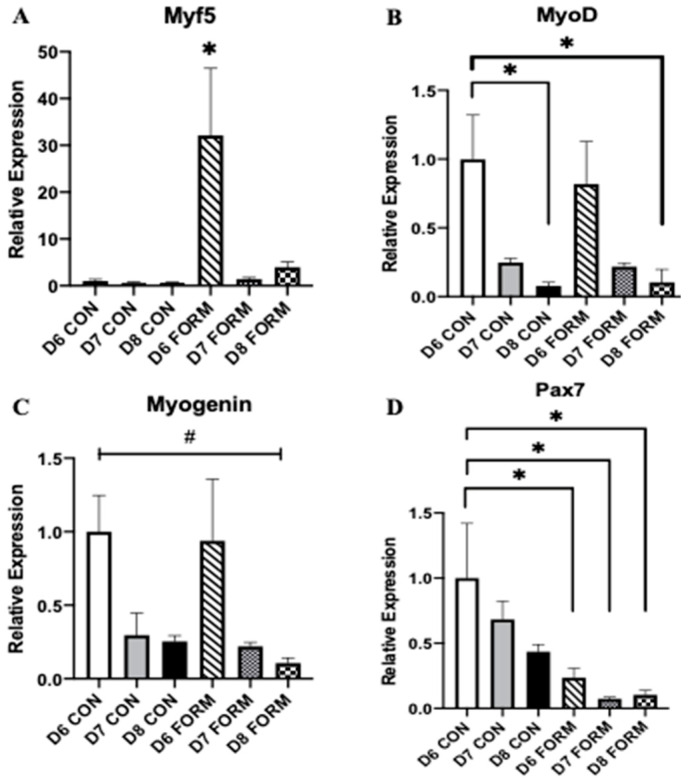
For each figure (n = 5), the symbol (*) is used to represent a significance difference between conditions and the symbol (#) is used to represent an overall significant effect for the ANOVA, with no significant differences between conditions. Myf5 (**A**): D6 FORM was increased compared to all other conditions (*p* < 0.05). MyoD (**B**): D8 CON was decreased compared to D6 CON (*p* = 0.025); D8 FORM was decreased compared to D6 CON (*p* = 0.032). Myogenin (**C**): There was an overall significant effect observed (*p* = 0.048; F = 2.775). Pax7 (**D**): D6 FORM was decreased compared to D6 CON (*p* = 0.044); D7 FORM was decreased compared to D6 CON (*p* = 0.008); D8 FORM was decreased compared to D6 CON (*p* = 0.011).

Myogenesis is tightly regulated by a series of transcription factors, including Myf5, MyoD, Myogenin, and Pax7. At day 6, Myf5 expression was increased in response to acute formoterol stimulation (D6 FORM; see Figure 1A). There were no other changes in Myf5 expression in response to two (D7 FORM) or three consecutive (D8 FORM) days of formoterol stimulation. At day 8, MyoD expression was decreased for D8 FORM and D8 CON compared to D6CON (Figure 1B). There were no other observed changes in MyoD expression. There was an overall significant change in Myogenin expression; however, post-hoc testing resulted in no differences between conditions (Figure 1C). Pax7 expression was decreased in response to both acute (D6 FORM) and consecutive (D7 FORM and D8 FORM) days of formoterol stimulation compared to D6 CON (Figure 1D).

### 2.2. Formoterol Influences the Expression of Regulators of Skeletal Muscle Mass

**Figure 2:**
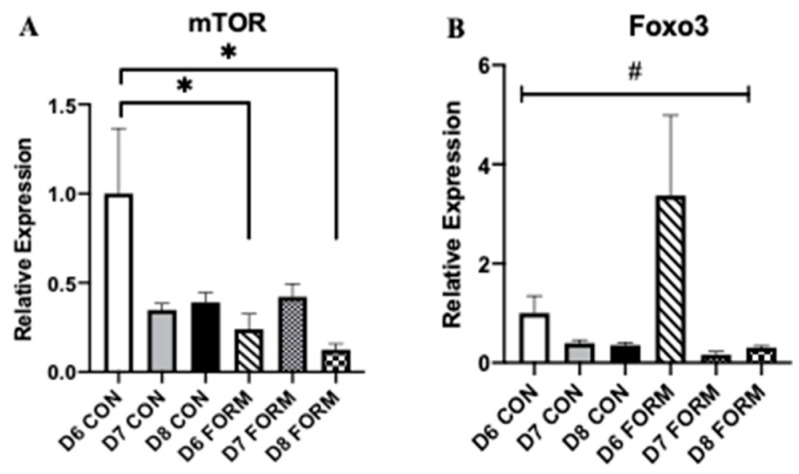

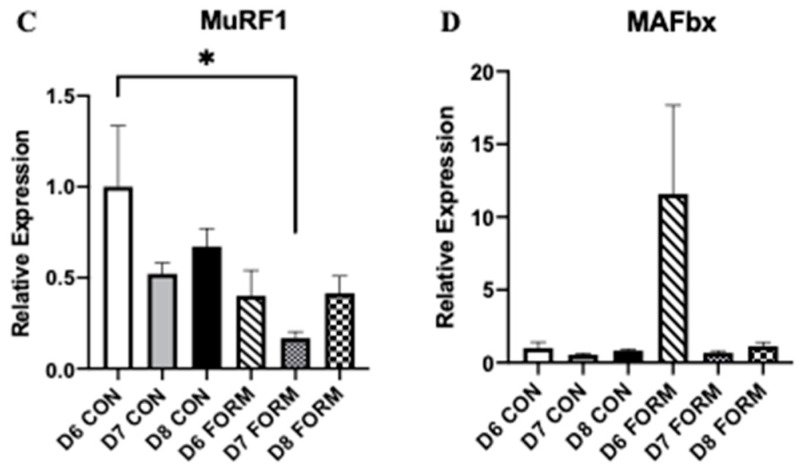
For each figure (n = 5), the symbol (*) is used to represent a significant difference between conditions and the symbol (#) is used to represent an overall significant effect for the ANOVA, with no significant differences between conditions. mTOR (**A**): D6 FORM was decreased compared to D6 CON (*p* = 0.015); D8 FORM was decreased compared to D6 CON (*p* = 0.004). Foxo3 (**B**): There was an overall significant effect observed (*p* = 0.006; F = 4.397). MuRF1 (**C**): D7 FORM was decreased compared to D6 CON (*p* = 0.029). MAFbx (**D**): There were no changes in expression between conditions.

Skeletal muscle mass and growth are regulated by several enzymes and transcription factors, with mTOR, Foxo3, MuRF1, and MAFbx having primary roles. mTOR expression was decreased following acute (D6 FORM) and consecutive (D8 FORM) days of formoterol stimulation compared to D6 CON (Figure 2A). No other changes in mTOR expression were observed. There was an overall significant change in Foxo3 expression; however, post-hoc testing resulted in no differences between conditions (Figure 2B). MuRF1 expression was decreased in response to two consecutive days (D7 FORM) of formoterol stimulation compared to D6 CON (Figure 2C). No changes in MAFbx expression were observed in this study (Figure 2D).

### 2.3. Formoterol’s Effects on Gene Expression Related to Mitochondrial Biogenesis and Beta-2 Adrenergic Receptors

**Figure 3:**
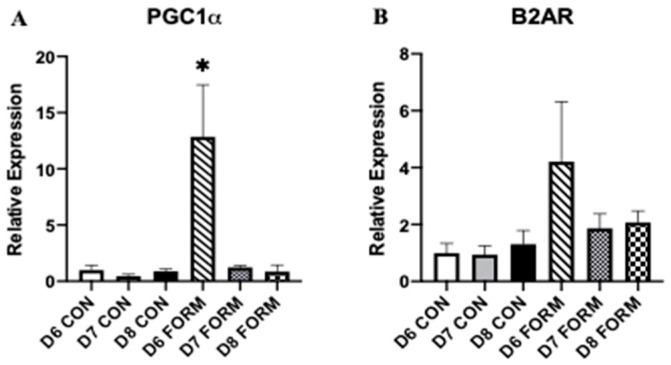
For each figure (n = 5), the symbol (*) is used to represent a significant difference between conditions. PGC-1α (**A**): D6 FORM was increased compared to all other conditions (*p* < 0.05). B2AR (**B**): There were no changes in expression between conditions.

Expression of PGC-1α, considered to be the master regulator of mitochondrial biogenesis, and beta-2 adrenergic receptor (B2AR), a membrane protein responsible for inducing formoterol’s intracellular effects, were measured, as both have important functions related to cell activity and mitochondrial biogenesis within skeletal muscle. PGC-1α expression was increased in response to acute formoterol stimulation (D6 FORM) compared to all other conditions (Figure 3A). No other changes in PGC-1α expression were observed. No changes in B2AR expression were observed in this study (Figure 3B).

### 2.4. Formoterol Stimulates and Influences the Gene Expression of miR-206

**Figure 4:**
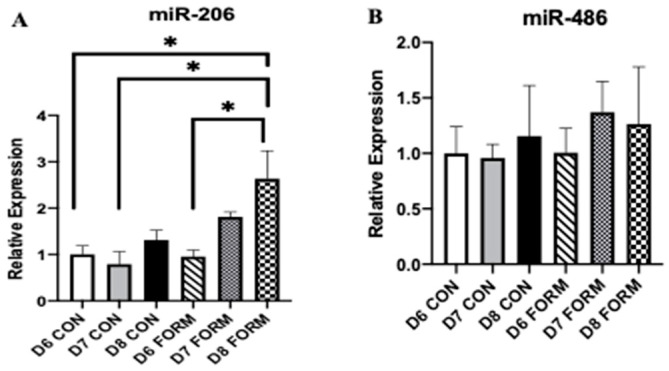
For each figure (n = 5), the symbol (*) is used to represent a significance difference between conditions. miR-206 (**A**): D8 FORM was increased compared to D6 CON (*p* = 0.011); D8 FORM was increased compared to D7 CON (*p* = 0.003); D8 FORM was increased compared to D6 FORM (*p* = 0.009). miR-486 (**B**): There were no changes in expression between conditions.

Due to their regulatory functions on skeletal muscle myogenesis and growth, we measured gene expression of miR-206 and miR-486. miR-206 expression was increased in response to consecutive days of formoterol stimulation (D8 FORM) compared to D6 CON, D7 CON, and D6 FORM (Figure 4A). No changes in miR-486 expression were observed (Figure 4B).

## 3. Discussion

We used an in vitro cell culture model to investigate how exercise-related signaling affects the expression of miRNA and genes related to myogenesis, muscle growth and mass, and cell and mitochondrial function within human skeletal muscle during late-stage myogenesis. Myogenesis is primarily regulated by MRFs [18]. Each individual MRF governs a specific phase(s) of myogenesis (early-, mid-, and late-stage myogenesis); however, the MRFs also operate collectively, with some MRFs having overlapping functions that promote the maturation and development of skeletal muscle [19,20]. Within this study, we found MRF expression varied in response to acute and consecutive days of formoterol stimulation. Expression of Myf5, a MRF principally involved during early-to-mid-stage myogenesis, was increased in D6 FORM. Myf5 expression was also increased in D7 FORM and D8 FORM; however, neither reached statistical significance. Importantly, our results for Myf5 mirror previous observations from our lab, whereby Myf5 expression peaked in response to formoterol stimulation on day 6 of differentiation (in review). In comparison to Myf5, expression of MyoD and Myogenin expression was decreased in response to acute formoterol stimulation, and this expression was further decreased with consecutive days of formoterol stimulation. Considering Myf5, MyoD, and Myogenin expression in the present study (Figure 2A–C), a pattern emerges, whereby expression of all three MRFs in D6 FORM is noticeably elevated compared to D7 FORM and D8 FORM. Initially, it appears that consecutive days of formoterol stimulation may have deleterious effects on MRF expression beyond day 6. However, we observed similar trends for Myf5, MyoD, and Myogenin in D7 CON and D8 CON, which implies that, beyond day 6 of differentiation, myogenic activity of skeletal muscle may be reduced. Despite this, the increase in Myf5 expression in D6 FORM suggests that formoterol may promote the myogenic process. As Myf5 expression is typically highest during early-to-mid myogenesis, we speculate that, had experiments been carried out over a longer period (hours/days following formoterol stimulation), there may have been additional changes observed in the expression of MyoD and Myogenin.

Secondary to formoterol’s stimulatory effects, we believe myomiR expression, specifically miR-206, could have potentially influenced our observations for Myf5 in response to consecutive days of formoterol stimulation. miR-206 promotes myogenesis through the inhibition of several targets (Pax7, HDAC4) during early-to-mid myogenesis [7,10,21]. Presently, we found that miR-206 expression increased with consecutive days of formoterol stimulation, with its expression being significantly elevated in D8 FORM. This increase in miR-206 expression in D8 FORM occurs simultaneously with increased Myf5 expression in D8 FORM (though this was not statistically significant). Thus, it appears that miR-206 expression is responsive to multiple consecutive days of formoterol stimulation, and this increase in miR-206 expression may be tied to myogenic activity within skeletal muscle; however, more work is necessary to delineate this relationship. Our observations for Myf5 may also be explained by examining B2AR expression in response to acute and consecutive days of formoterol. B2AR serves as the primary receptor for formoterol, permitting its effects intracellularly [22]. Presently, patterns for B2AR expression in D6 FORM, D7 FORM, and D8 FORM (Figure 4B) were similar to those observed for Myf5, MyoD, and Myogenin. B2AR expression is noticeably elevated in D6 FORM compared to D7 FORM and D8 FORM, though this did not reach statistical significance. From this, we theorize that, following the first day of formoterol stimulation, desensitization through agonist-receptor binding may have occurred, minimizing subsequent downstream intracellular effects that are observed in D7 FORM and D8 FORM.

Within this study, we also investigated formoterol’s effects on regulators of skeletal muscle mass and growth. Growth of skeletal muscle is regulated by a complex and interconnected network of mechanical, hormonal, and chemical stimuli. Central to this is mTOR due to its regulation of protein synthesis [23,24]. Several studies using animal models have reported formoterol can induce mTOR expression, and this is likely achieved through formoterol’s activation of the PI3K/Akt pathway [25,26,27]. We observed reductions in mTOR expression in D6 FORM and D8 FORM, suggesting that acute and consecutive days of formoterol stimulation may inhibit molecular regulation of skeletal muscle growth. The observations for mTOR may be explained by the increase in miR-206 expression. Despite miR-206′s effects on myogenesis, it appears to have an inhibitory role on skeletal muscle growth, targeting Akt and insulin-like growth factor 1 (IGF-1), both of which are involved in mTOR activation [10,28]. Thus, by increasing miR-206 expression, both acute and consecutive days of formoterol stimulation may have blunted mTOR expression. In addition to miR-206, we also measured the expression of miR-486, which appears to indirectly promote skeletal muscle growth through its inhibition of phosphatase and tensin homolog (PTEN), as well as its targeting of FoxO1 and FoxO3 [7,10,28]. Despite this, we did not observe any significant changes in miR-486 expression with either acute or consecutive days of formoterol stimulation. Worth mentioning, although a mechanistic link between the beta-2 adrenergic receptor and the PI3K-Akt-mTOR signaling pathway has been established [27], our observations for mTOR expression in response to formoterol stimulation mirror previous findings from our laboratory (in review).

Our observations for mTOR may also be explained by our findings for PGC-1α, FoxO3, and MAFbx. Previous studies have demonstrated that formoterol is a robust stimulator of PGC1α expression within skeletal muscle myoblasts [29,30]. Presently, we observed a significant increase in PGC-1α expression in D6 FORM, which suggests that acute formoterol stimulation may have preferentially shifted cell and molecular activity towards a metabolically demanding state, minimizing any potential effect formoterol may have had on regulators of skeletal muscle growth. In addition, we observed increases in FoxO3 and MAFbx expression in D6 FORM, though neither was statistically significant compared to other groups. Considering the increase in PGC-1α expression in D6 FORM, the observations for FoxO3 and MAFbx may have been a consequence formoterol’s potential effects of shifting cell and molecular activity to metabolic regulation. However, we did observe a decrease in MuRF1 expression with in D7 FORM, suggesting that, beyond acute exercise stimulation, consecutive days of formoterol stimulation may attenuate muscle protein degradation. The mechanisms explaining these potential interactions between acute and consecutive days of formoterol stimulation and factors involved in skeletal muscle growth and muscle protein degradation are unknown and require further investigation. Additionally, investigations exploring the expression of genes associated with metabolic and aerobic capacity of skeletal muscle cells (e.g., AMPK) in consideration of our results for PGC-1α are warranted to further explain our observations in this study.

## 4. Materials and Methods

### 4.1. Cell Culture and Myotube Formation

**Figure 5:**
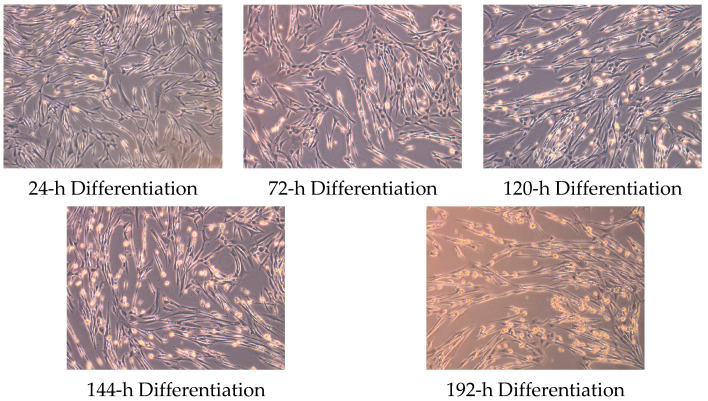
Skeletal muscle cells at various stages of differentiation.

Primary human skeletal muscle myoblasts obtained from healthy adult donors (HSkMC 150-05A, passage 4-6; Sigma-Aldrich, St. Louis, MO, USA) were cultured in 35 mm 6-well collagen coated plates (Gibco, New York, NY, USA) at 37 °C in 5% CO_2_ in skeletal muscle cell growth media (151-500 Sigma-Aldrich). Myoblasts (n = 5) were seeded at a density of 80,000 cells per well. At confluency (80–90%), differentiation medium (151D-250 Sigma-Aldrich) was used to initiate myotube formation. Differentiation media was replaced every 48 h, and differentiation was achieved within ~6 days. Images representing progressive stages of myoblast differentiation are shown in Figure 5.

### 4.2. Exercise Mimetic

Formoterol fumarate dehydrate > 98% HPLC (F9552 Sigma-Aldrich), reconstituted in dimethyl sulfoxide (DMSO) and mixed into differentiation medium at a concentration of 30 nM. DMSO, was used as a control by adding a volume equal to formoterol to the differentiation media for the control group in this study. Three hours prior to scheduled RNA extraction in each experimental condition, differentiation media were changed to either fresh control media or formoterol-treated media for the remaining duration (i.e., three hours) of the incubation period.

### 4.3. Experimental Conditions

**Figure 6:**
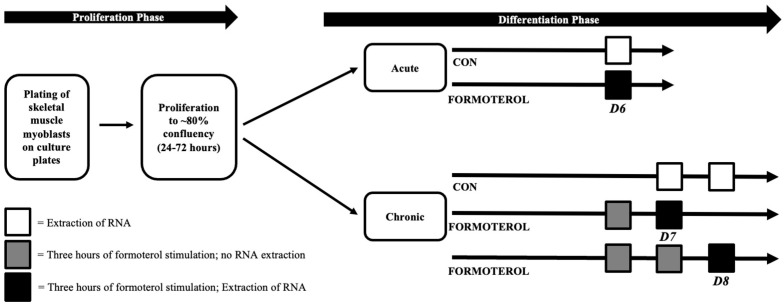
Overview of study timeline and methodology.

Three experimental conditions were utilized in this study: (a) control (CON), (b) acute formoterol stimulation (AFS), and (c) consecutive days of formoterol stimulation (CFS). For AFS, mature myotubes were treated with 30 nM of formoterol for three hours on day 6 of differentiation (D6), and this was immediately followed by RNA extraction. Similarly, for CFS, mature myotubes were treated with 30 nM of formoterol for three hours on two (D7) or three consecutive days (D8). RNA was extracted immediately following the final three-hour formoterol treatment for D7 and D8. On days in which D7 and D8 received formoterol, but RNA was not extracted, formoterol media was removed, culture wells were washed with PBS (Gibo, New York, NY, USA), and fresh differentiation media was added. Respective CON conditions (D6 CON, D7 CON, D8 CON) for each treatment condition were also included to make comparisons between AFS and CFS. Figure 6 provides an overview of the methodology used in this study.

### 4.4. RNA Extraction

Cells were extracted on D6, D7, and D8 of myogenesis. Total RNA was extracted according to the manufacturer’s instructions using a Qiagen miRNeasy Kit (Qiagen, Germantown, MD, USA) and stored for further analysis at −80 °C.

### 4.5. Gene Expression of Markers Related to Myogenesis, Muscle Growth, and Mitochondrial Actvitiy

Complementary DNA was synthesized from 1μg of the resulting total RNA using a High Capacity cDNA Reverse Transcription Kit (Applied Biosystems, Carlsbad, CA, USA). Real-time quantitative polymerase chain reaction (qPCR) detection was performed in duplicate using PowerUp SyBR and QuantStudio RealTime 3 PCR System (Applied Biosystems, Carlsbad, CA, USA). Forward and reverse primers for each target gene (Integrated DNA Technologies, Coralville, IA, USA) were used, and data were analyzed using the comparative Ct (ΔΔCt) method for quantification. Ribosomal protein S13 (RPS13) was used as the endogenous control for comparative data of target genes. Forward and reverse primer sequences used for assessing gene expression of gene targets, as well primer sequences for targeted miRNA, are provided in Table 1.

### 4.6. Gene Expression of miRNA

15 μL reverse transcriptase-polymerase chain reactions were generated using a TaqMan™ Small RNA Assays Kit (Applied Biosystems, Carlsbad, CA, USA) in a Bio-Rad T100 Thermocycler (Bio-Rad Laboratories, Hercules, CA, USA). All reactions used TaqMan™ Fast Advanced Master Mix (ThermoFisher, Waltham, MA, USA) and real-time qPCR was performed in duplicate using QuantStudio RealTime 3 PCR System. Target miRNA (miR-206 and miR-486) were compared with an established candidate control gene, miR-92a, to determine fold changes in gene expression.

### 4.7. Statistical Analysis

Differences in gene expression in this study were analyzed using SPSS v25 (IBM, Armonk, NY, USA). One-way repeated measures analysis of variance (ANOVA) was used to determine significant differences between experimental conditions, and Bonferroni post-hoc testing was used to make pairwise comparisons between groups. Statistical significance was established at *p* < 0.05.

## 5. Conclusions

In the present study, we utilized an in vitro cell culture model to determine how genes regulating myogenesis and skeletal muscle growth are influenced in response to both acute and consecutive days of exercise-related stimulation using the exercise mimetic, formoterol, in human skeletal muscle. A secondary aim of this study was to examine how miR-206 and miR-486, myomiRs, which regulate both myogenesis and skeletal growth, respond to both acute and consecutive days of formoterol stimulation, while also investigating how miR-206 and miR-486 may influence genes that regulate both myogenesis and skeletal growth. We found that acute and consecutive days of formoterol stimulation increased miR-206 and had no effect on miR-486 expression. Additionally, we found that acute formoterol stimulation promoted myogenesis, increasing the expression of Myf5, but this effect was lost with consecutive days of formoterol stimulation. Lastly, we observed increased expression of genes involved in metabolic and mitochondrial activity, as well as muscle protein degradation in response to acute formoterol stimulation. These effects were no longer observed with consecutive days of formoterol stimulation, and interestingly gene expression representative of muscle protein synthesis was reduced with both acute and consecutive days of formoterol stimulation.

Collectively, the results from this study provide an interesting framework for the interconnected network between miRNA and genes regulating myogenesis and skeletal muscle growth in response to exercise stimulation. Additional in vitro and in vivo research addressing this relationship, and the nuances between acute and long-term exercise on these processes, is needed to characterize the importance of miRNA on skeletal muscle physiology in greater detail.

## Figures and Tables

**Table 1:** Primer sequences used for assessing gene and miRNA expression.

Gene	Primer	Sequence (5′-3′)
Myf5	Forward	GCTTCTAGTTCCAGGCTTATC
	Reverse	GCCTTCTTCTTCCTGTGTATTA
MyoD	Forward Reverse	CACAACGGACGACTTCTATG GTGCTCTTCGGGTTTCAG
Myogenin	Forward Reverse	CCCTGAATTGAGAGAGAAGAAG CGGATGGCAGCTTTACAA
Pax7	Forward Reverse	GAAGACGAAGAAGACGGAAAG GGACACTTCCAAAGGGAATC
mTOR	Forward Reverse	GGACTACAGGGAGAAGAAGA CATCAGAGTCAAGTGGTCATAG
Foxo3	ForwardReverse	CCACCCTTGGCCTCTAAATAA GGTAACAGGTATCAGGTTCTGG
MuRF1	Forward Reverse	CAGCTGGACAAGTCCACAAA GCGTCTGCTATGTGCTCTAAAT
MAFbx	Forward Reverse	GCATGCCCTTGGCAAATAAG ATGTGGGTTGTGTGCTATTGA
PGC-1α	Forward Reverse	TCTCTCTCTCTCTCTCTCTCT CATGGGTGTCAGGATTAAGG
B2AR	Forward Reverse	CCTGCTGACCAAGAATAAGG GCAGGTCTCATTGGCATAG
RPS13	Forward Reverse	GCATCTTGAGAGGAACAGAAA AGGACTCGCTTGGTCTTAT
hsa-miR-206	Mature Sequence	UGGAAUGUAAGGAAGUGUGUGG
hsa-miR-486	Mature Sequence	UCCUGUACUGAGCUGCCCCGAG
hsa-miR-92a	Mature Sequence	UAUUGCACUUGUCCCGGCCUGU

## Data Availability

The data used and/or analyzed in this current study are available from the corresponding author upon reasonable request.
